# Sickness Amongst Nurses

**Published:** 1921-03-12

**Authors:** 


### Sickness amongst Nurses.

The Health Committee of the Willesden Urban District Council reports that twelve out of a staff of fifty nurses and five out of a staff of thirty-seven maids are at present off duty owing to indisposition, but despite this handicap their remaining staff are giving the medical superintendent their loyal assistance in carrying out their duties at the Municipal Hospital. The medical superintendent has now been obliged to engage one private nurse at £3 16s. 6d. a week, and five private nurses at £2 12s. 6d. each.

